# Supplementary figures and images for: Complete De Novo Assembly of Wolbachia Endosymbiont of Frankliniella intonsa
Source: Int J Mol Sci. 2023 Aug 26;24(17):13245. doi: 10.3390/ijms241713245 (PMC10487741; doi:10.3390/ijms241713245)

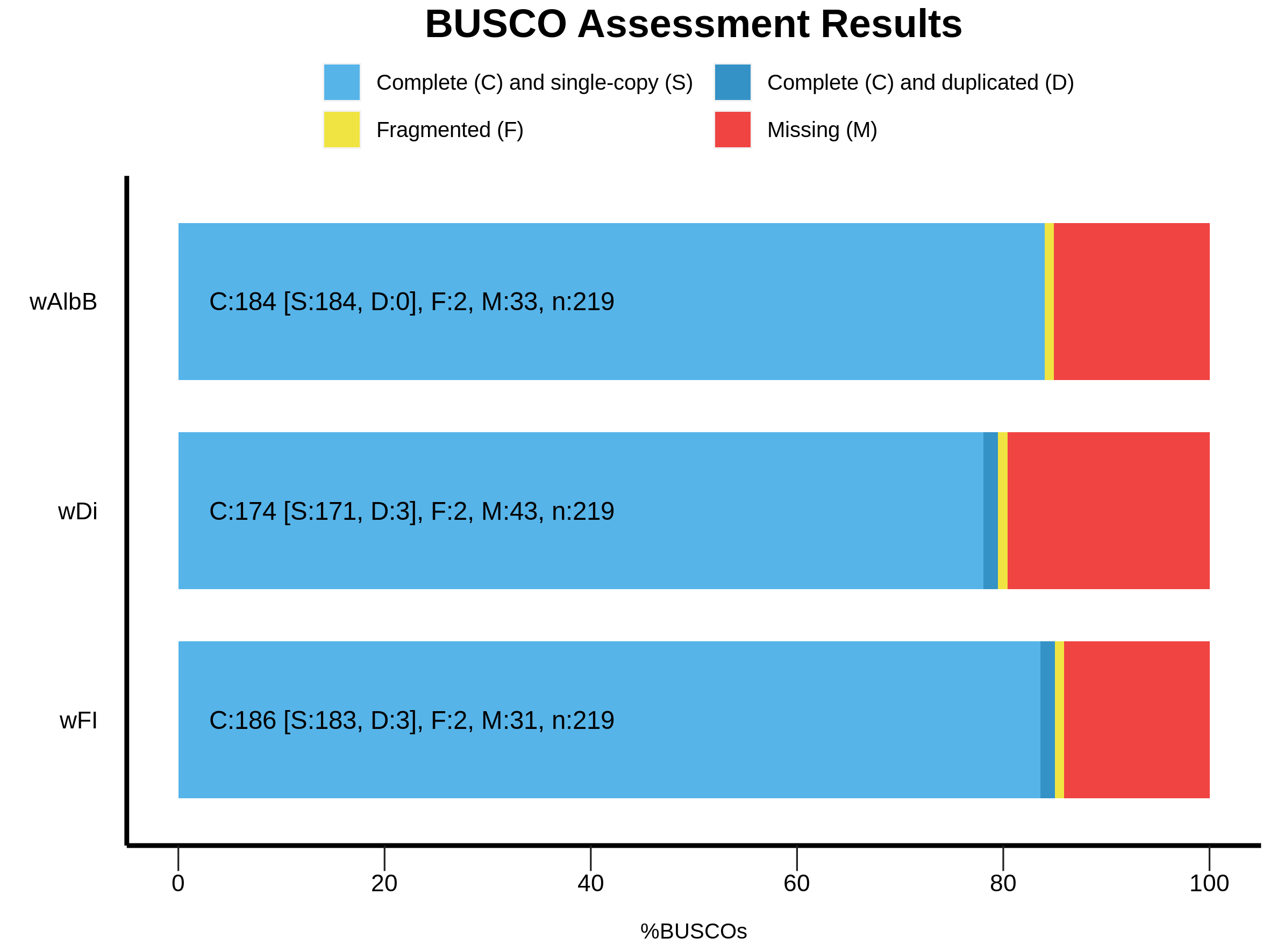

Supplement: Supplementary file 1 [file ijms-24-13245-s001.zip › ijms-2546802-supplementary/Supplementary_Material/Figure S1.png]

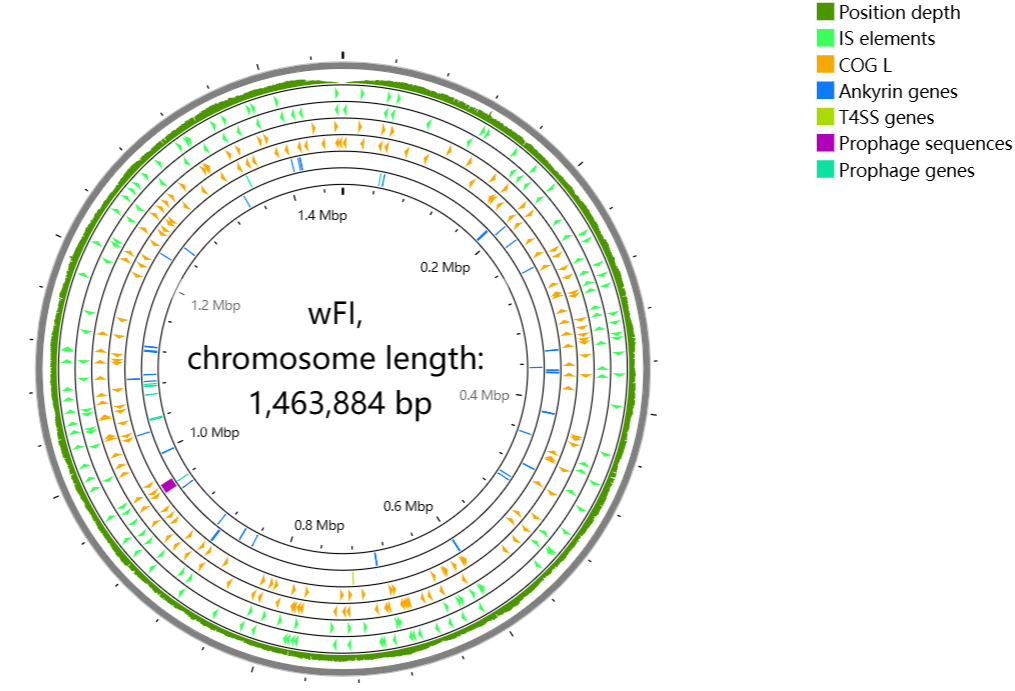

Supplement: Supplementary file 1 [file ijms-24-13245-s001.zip › ijms-2546802-supplementary/Supplementary_Material/Figure S2.png]

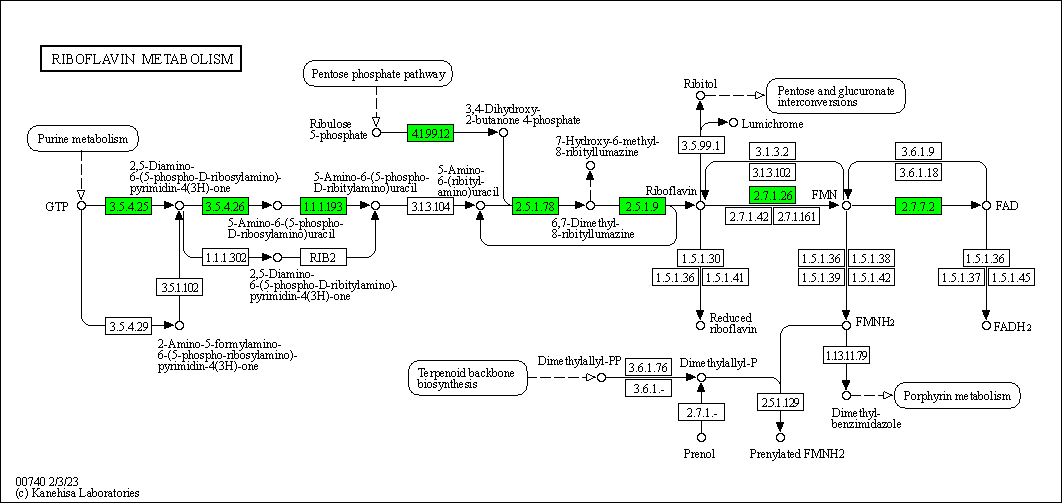

Supplement: Supplementary file 1 [file ijms-24-13245-s001.zip › ijms-2546802-supplementary/Supplementary_Material/Figure S3.png]

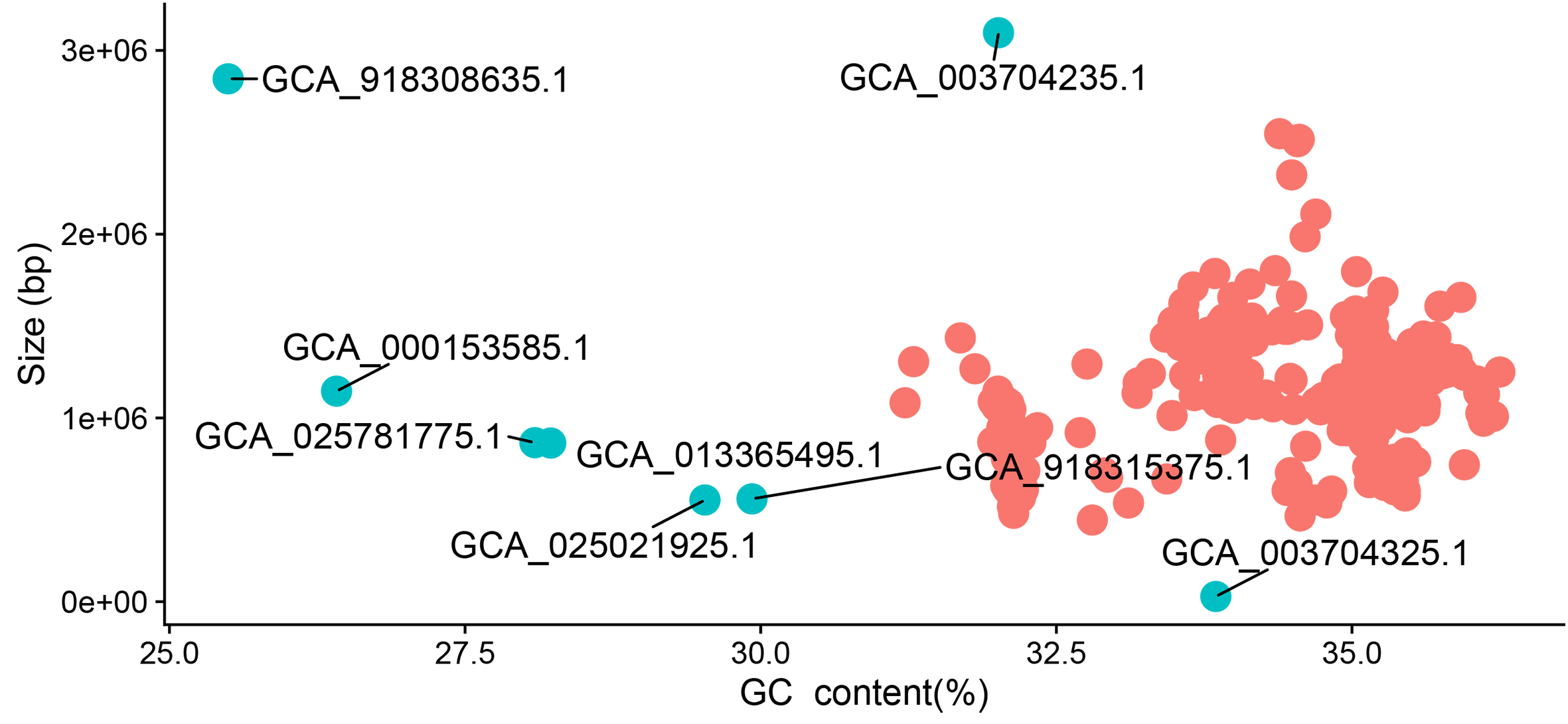

Supplement: Supplementary file 1 [file ijms-24-13245-s001.zip › ijms-2546802-supplementary/Supplementary_Material/Figure S4.tif]

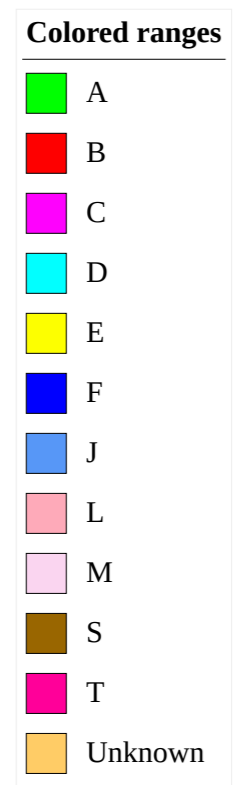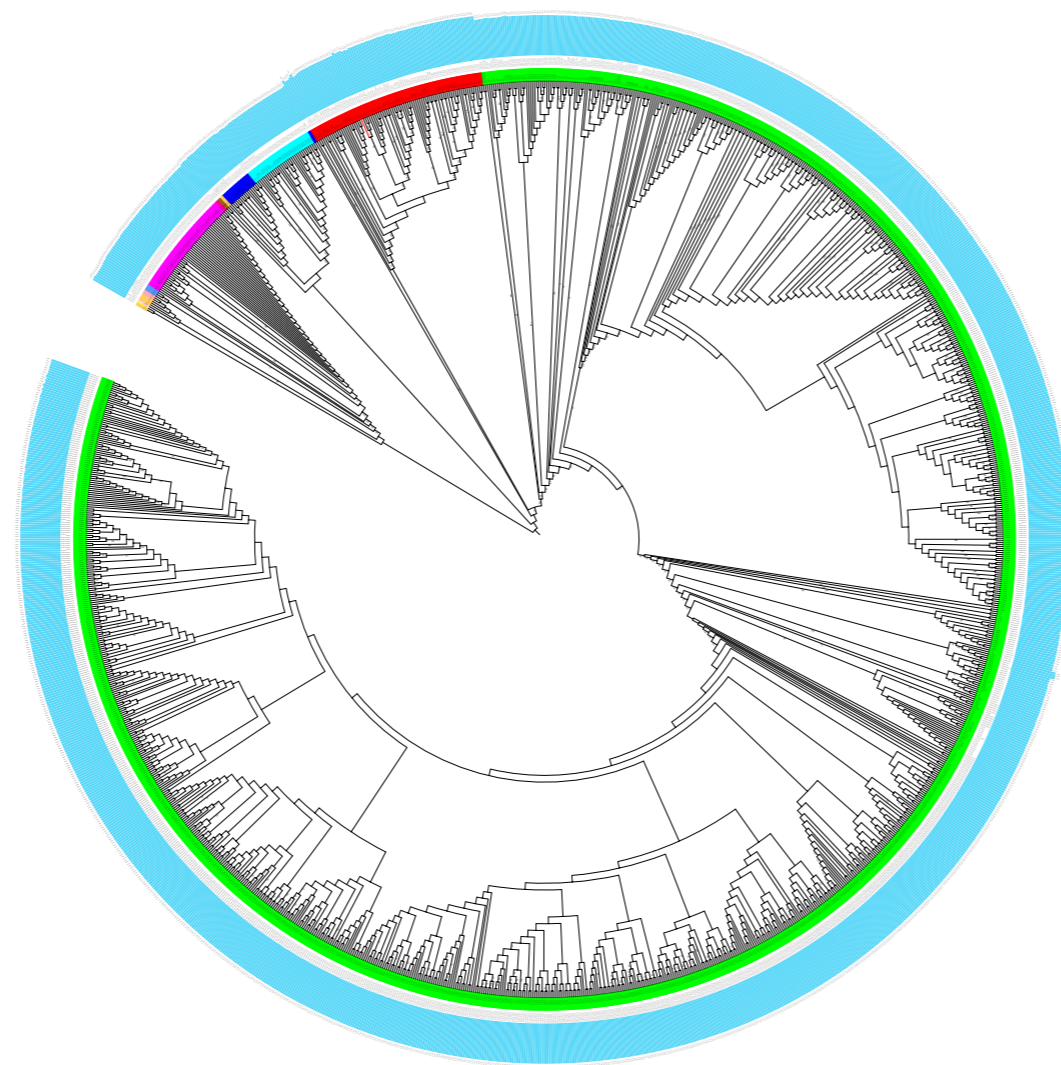

Supplement: Supplementary file 1 [file ijms-24-13245-s001.zip › ijms-2546802-supplementary/Supplementary_Material/Figure S5.pdf]
